# Magnesium sulphate attenuate remifentanil-induced postoperative hyperalgesia via regulating tyrosine phosphorylation of the NR_2_B subunit of the NMDA receptor in the spinal cord

**DOI:** 10.1186/s12871-017-0325-3

**Published:** 2017-02-21

**Authors:** Jiehao Sun, Hai Lin, Guodong He, Wendong Lin, Jianping Yang

**Affiliations:** 1 Department of Anesthesiology, 1st Affiliated hospital, Soochow University, 188#, Shizi Street, Gusu District, Suzhou, 215000 China; 2 Department of Anesthesiology, 1st Affiliated Hospital of Wenzhou Medical University, Wenzhou, China

**Keywords:** Magnesium, Remifentanil, Hyperalgesia, NMDA receptor, NR_2_B

## Abstract

**Background:**

Remifentanil induced hyperalgesia (RIH) is characterized by stimulation evoked pain including allodynia and thermal hyperalgesia after remifentanil infusion. N-methyl-D-aspartate (NMDA) receptor was reported to be involved in the progress of RIH. We hypothesized that intrathecal MgSO_4_ could relieve hyperalgesia after remifentanil infusion via regulating phosphorylation of NMDA receptor NR_2_B subunit activity in this study.

**Methods:**

Thirty two rats were randomly allocated into control group, model of RIH group, RIH plus 100ug MgSO_4_ group, RIH plus 300ug MgSO_4_ group. Mechanical and thermal hyperalgesia were tested at -24^th^ h, 2^nd^ h, 6^th^ h, 24^th^ h, 48^th^ h after remifentanil infusion. Following sacrifice of rats after the last behavioral test, we performed the western blot to detect the expression of spinal phosphorylated NMDA receptor NR_2_B subunit (pNR_2_B) in the L_4_-L_5_ segments.

**Results:**

Intrathecal MgSO_4_ (100, 300 μg) dose-dependently reduced thermal and mechanical hyperalgesia from 2 h to 48 h after remifentanil infusion. Remifentanil infusion remarkably stimulated the expression of pNR_2_B. Nevertheless, the increased amount of pNR_2_B by RIH was dose-dependently suppressed by intrathecal infusion of MgSO_4_ in rats.

**Conclusions:**

Remifentanil induced hyperalgesia/allodynia could be ameliorated by Mg-mediated blockade targeting the NR_2_B subunit in NMDA receptors.

**Electronic supplementary material:**

The online version of this article (doi:10.1186/s12871-017-0325-3) contains supplementary material, which is available to authorized users.

## Background

Due to the property of rapid onset and recovery, remifentanil is quite widely used in clinic. However, remifentanil was reported to be associated with the development of hyperalgesia. it was noted that general anesthesia based on remifentanil infusion resulted in severe postoperative pain after surgery [1]. Additionally, analgesic consumption after operation can be increased following the intraoperative use of remifentanil [2].

N-methyl-D aspartate (NMDA) receptors was known to play a critical role in excitatory synaptic transmission. Remifentanil could enhance the activation of spinal NMDA receptor, which was attributed to the progress of remifentanil induced hyperalgesia (RIH) [3].

NR_2_B subunit is an essential part in the NMDA receptors. Tyrosine-1472 phosphorylation in NR_2_B is known to be associated with neuropathological conditions [4]. Phosphorylation of Tyr-1472 in NR_2_B (pNR_2_B) has previously been demonstrated in the progress of RIH [5].

NMDA receptor antagonist was implicated to decrease the extended area of RIH in human [6]. Prior administration of magnesium could inhibit delayed fentanyl induced hyperalgesia [7, 8] in rats. Our previous study suggested that magnesium sulphate could dose-dependently inhibit RIH in rats [9]. The purpose of this study was to explore whether intrathecal magnesium administration could prevent RIH via decreasing the amount of pNR_2_B expression in superficial dorsal horn of spinal cord.

## Methods

### Animals

After approval of experimental animal center of Wenzhou Medical university, Adult male Sprague–Dawley rats (230 ± 30 g) were obtained and maintained under a 12 h light −12 h dark cycle with food and water freely available.

### Intrathecal catheter placement

Intrathecally catheterization was performed after an acclimation period of at least 5 days for the rats. Under sevoflurane anesthesia, plastic PE-10 tube (OD: 0.5 mm, ID: 0.25 mm, AniLab Co. Ltd, China) was implanted into intrathecal space. The rostral part of the catheter was then sutured to the muscle to immobilize it.

### Surgical procedure

Under sevoflurane anesthesia, a longitudinal incision (0.8 cm) was made starting from edge of the heel and extending toward the toes of the right hind paw. The underlying plantaris muscle was exposed and incised longitudinally, leaving the muscle origin and insertion intact. After hemostasis, skin was closed covered with antiseptic gauze.

### Remifentanil infusion

Remifentanil (1.2 μg.kg^−1^.min^−1^) was infused via tail vein over a period of 60 min using a pump.

### Behavioral test

Mechanical nociception was determined by measuring paw withdrawal mechanical thresholds (PWMT). Through a mesh bottom (1 × 1 cm), the electronic von Frey filament (0.8 mm diameter, LS instrument, USA) was applied vertically to the plantar surface of the right hind paw. Positive nociceptive-like response was defined as clear paw withdrawal or licking.

Thermal nociception was determined by measuring paw withdrawal thermal latency (PWTL) using thermal stimulation system (Model 336, Series 8, IITC INC, USA). A radiant thermal source below a glass floor (5 mm thick) was positioned to deliver a thermal stimulus to the midplantar region adjacent to the wound of right hind paw. When the rat had response of clear paw withdrawal or flinching, the thermal source was switched off, and the timer stopped, measuring the PWTL. Thermal stimulation was automatically cut off after 25 s if the rat fails to withdraw.

Animals were allowed to acclimatize for 30 min before testing. Mean PWTL and PWMT were established by averaging the values of three tests with a five minute interval between each test.

### Drugs

Remifentanil hydrochloride (Ultiva) (batch number: 6587129, Ren Fu Co, China), Magnesium sulfate (M2643, Sigma. St. Louis, USA), sevoflurane (batch number: 4Z132, maruishi-pharm.co. Japan).

### Western blotting

After the last behavioral test, the animals were sacrificed with sevoflurane and lumbar spinal cord L_4_-L_5_ segments were removed in 2 min. Tissue samples were homogenized in lysis buffer containing protease inhibitors (Sigma-Aldrich Co.). The homogenate was centrifuged at 12,000 rpm for 5 min at 4 °C and supernatant was removed as the total protein. Proteins (70 μg) were separated on a 7.5% sodium dodecyl sulfate–polyacrylamide gel electrophoresis (SDS-PAGE) and transferred to polyvinylidene difluoride membrane (Bio-Rad, CA) with a Trans-Blot Transfer Cell system (Bio-Rad, CA). The filters were blocked with 5% nonfat milk in TBS buffer at room temperature for 1 h. Then the blot was incubated with the primary antibody against phosphorylated Tyr 1472 NR_2_B (1:1000; Cell signaling Technology, USA) overnight at 4 °C. The membrane was washed with TBS buffer and incubated for 1 h with the secondary anti- rabbit IgG horseradish peroxidase (1:2000; Jackson ImmunoResearch, USA) at room temperature and visualized in enhanced chemiluminescence solution (Amersham Biosciences) followed by film exposure. β-Actin was used as endogenous control (1:10,000, Biotechnology, USA). Those who did the western blot were blinded to the group allocation. Densitometric quantification of each specific band was performed using Gene Tools Match software (Syngene, Cambridge, UK). The results were expressed as the percentage of β-actin immunoreactivity.

### Experiment protocol

Experiments were performed 7 days later after intrathecal catheterization. To evaluate thermal and mechanical hyperalgesia induced by remifentanil, 32 rats were assigned into the following four experimental groups (*n* = 8): 1: Group C (a control group with the administration of sevoflurane inhalation without incision); 2: Group RI (model of RIH with surgical procedure and remifentanil infusion, 10 μl normal saline was intrathecally administration); 3: Group RIM_3_ (300 μg MgSO_4_ was intrathecally given to the group RI); 4: Group RIM_1_ (100 μg MgSO_4_ was intrathecally given to the group RI). 30 min before remifentanil infusion and plantar incision, MgSO_4_ or normal saline were intrathecally administration in a volume of 10 μl, followed by additional normal saline (20 μl) to flush the catheter. Remifentanil infusion and plantar incision were performed at the same time. PWMT and PWTL tests were performed at −24 h, 2 h, 6 h, 24 h, and 48 h after remifentanil infusion. The L_4_-L_5_ segments for western blot analysis were collected just after behavioral testing at 48 h to investigate whether magnesium could prevent RIH via modulating spinal pNR_2_B activity.

All behavioral tests were carried out in a quiet test room by the same investigator (Doc Lin) who was blinded to the group allocation.

### Statistical analysis

Quantitative parametric data obtained from the groups were expressed as mean ± SD. Data from thermal and mechanical hyperalgesia were analyzed using repeated measures analysis of variance. The expression of pNR_2_B across all experimental groups were statistically tested by one-way ANOVA with Bonferroni correction. The level of statistical significance was set at *p* = 0.05. Data were analyzed with the SPSS 15.0 software (SPSS Inc., Chicago, IL, USA).

## Result

There were no statistical differences by the basal PWTL and PWMT values among the experimental groups (*p* > 0.05).

**Fig. 1:**
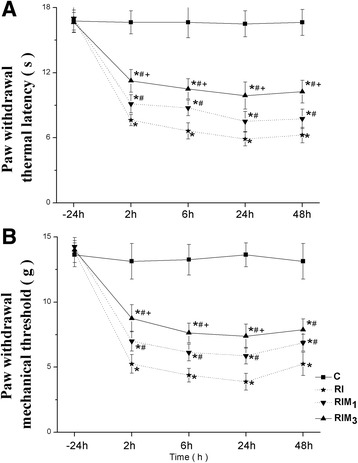
**a**-**b** Effect of MgSO_4_ on remifentanil-induced hyperalgesia. MgSO_4_ or normal saline were administered intrathecally 30 min before remifentanil infusion and surgical incision. PWTL (**a**) and PWMT (**b**) were evaluated at 24 h before incision and 2, 6, 24, 48 h. Data are expressed as means ± SD. * *p* < 0.001 compared with Group C, ^#^ *p* < 0.001 compared with Group RI, ^+^ *p* < 0.01 compared with GroupRIM_1_

Compared with normal saline, both of 300 μg and 100 μg MgSO4 significantly inhibited thermal (Fig. 1a) and mechanical (Fig. 1b) hyperalgesia. Compared with 100 μg MgSO_4_, 300 μg MgSO_4_ had less thermal hyperalgesia at 2 h, 6 h, 24 h, 48 h (*p* = 0.000) and less mechanical hyperalgesia at 2 h (*p* = 0.009),6 h (*p* = 0.005), 24 h (*p* = 0.004). There was no statistical difference in mechanical hyperalgesia at 48 h (*p* = 0.286) (Fig. 1, Additional file 1).

**Fig. 2:**
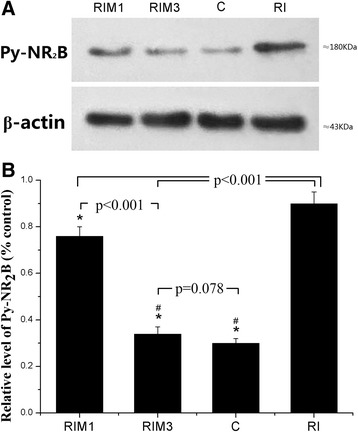
**a**-**b** The expression level of tyrosine phosphorylation of NR_2_B in superficial spinal cord in each group. L_4_-L_5_ segments of spinal cord were collected at 48 h after the remifentanil infusion. **a** Bands of Western blot for pNR_2_B. β-Actin is a loading control. **b** Quantification of pNR_2_B in each group. Data are expressed as means ± SD. * *p* < 0.001 compared with Group RI, ^#^ *p* < 0.001 compared with Group RIM_1_

The levels of tyrosine phosphorylated NR_2_B of NMDA receptors were significantly lower in GroupRIM_3_ compared with the levels in GroupRIM_1_ and Group RI (*p* < 0.001). No significant change was detected between GroupRIM_3_ and Group C (*p* = 0.078). (Fig. 2, Additional file 2).

## Discussion and conclusions

In the current study, remifentanil was found to aggravate both mechanical and thermal hyperalgesia after remifentanil infusion. Intrathecal Mg^2+^ provided a dose-dependent anti-nociceptive effect [10]. The authors have previously demonstrated that the MgSO_4_ doses of 100ug and 300 μg were safe for intrathecal injection in rats [9]. MgSO_4_ was reported to alleviate the hyperalgesia via inhibiting the tyrosine phosphorylation of NMDA receptor in dorsal horn of lumber spinal cord [11].

Remifentanil was report to induce dose-dependent hyperalgesia in human [2]. The hyperalgesic effect of opioid was complicated and was once seemed to be mediated by opioid receptors. However, hyperalgesia could still be provoked by opioid in mice in which μ, κ and δ opioid receptors were completely knocked out [12, 13]. On the contrary, infusing the opioid could still cause hyperalgesia instead of analgesia in these knockout mice [13]. Based on the results of these studies, it is supposed that opioid-induced hyperalgesia may be explained by mechanism other than opioid receptors.

MgSO_4_ in the trial was found to decrease the remifentanil induced hyperalgesia. RIH may be associated with NMDA activation via an intracellular pathway to up-regulate the function of NMDA activity [3]. MgSO_4_ could occlude the Ca^2+^ ion current, which resulted in a further release of glutamate and initiating a series of central sensitization to induce opioid-induced hyperalgesia [14, 15]. As illustrated by Angst’s study, even short-term administration of remifentanil could enhance hyperalgesia during withdrawal, while ketamine, the NMDA receptor antagonist, could abolish such hyperalgesic response [6]. NMDA antagonists could reverse opioid hyperalgesia by antagonizing NMDA receptors [16]. Taken together, NMDA palyed a key role in pathogenesis of RIH.

Distribution of NMDA receptor NR_2_B was demonstrated to almost limit in the superficial dorsal horn [17]. The spinal sensitization and increased nociceptive input may present due to the condition that noxious stimulus occurred within the superficial dorsal horn in the central primary afferent terminals. Subsequently, nociceptive substances such as glutamic acids activate the specific nociceptive neurons and create the pain impulses. Following such injury, the expression of NR_2_B subunit in superficial dorsal horn was detected to increase significantly [18].

Protein phosphorylation is a major mechanism for the regulation of receptor activity. Tyr-1472 phosphorylation of NR_2_B subunit is important for synaptic plasticity and the development of central sensitization [19]. Furthermore, tyr-1472 phosphorylation in the superficial dorsal horn of the spinal cord contributed to the progress of hyperalgesia in neuropathic pain [20, 21]. Abrupt withdrawal from opioid use could activate NMDA receptors and increase postsynaptic calcium concentrations [22]. NMDA antagonist was found to block increased Tyr1472 phosphorylation and inhibit remifentanil induced hyperalgesia [23].

Mg deficiency could facilitate NMDA receptor activation and long-term sensitization of the nociceptive pathways [11]. Previous study has showed that intrathecal administration of MgSO_4_ was beneficial in rats with NMDA receptor-dependent hyperalgesia [15].

Our findings support our hypothesis that NR_2_B tyrosine phosphorylation could be alleviated by NMDA antagonists. However, direct evidence of magnesium to the NR_2_B tyrosine phosphorylation and hyperalgesia remains to be further explored. It is the first study to demonstrate that magnesium could prevent RIH via decreasing Tyr-1472 phosphorylation in NMDA receptor.

Sevoflurane was previously proved to have no influence on nociceptive thresholds, so it was used for anesthesia and animal sacrifice in this study [24]. The doses of MgSO_4_ employed in the trial was based on the previous study [9]. Limitations:

1. NMDA receptors are positioned in either cellular membrane or cytoplasm. In the previous study, it was demonstrated that membrane trafficking of NR_2_B subunits in the spinal cord were significantly promoted after remifentanil infusion [25]. Due to inadequate funding, we only extracted the total protein for western blot analysis. In the further study, we should extract the membrane protein of dorsal horn to detect the amount of pNR_2_B subunits after magnesium intervention.
2. Sole surgical incision can also induce hyperalgesia after operation. Why the hyperalgesia in the paper was attributed to the remifentanil infusion? Previous report from our research have demonstrated that remifentanil infusion alone could trigger more severe hyperalgesia than surgical incision alone [9]. In the figure, the experiment indicated that MgSO_4_ could inhibit RIH with statistical difference. However, in the Group RIM_3_ and Group RIM_1_, the thresholds of the behavior tests in the Fig. 1 were found to have the same level compared with the thresholds in the Group surgical incision alone in the figure (shown as Additional file 1). In the incision-absent group, it is virtually impossible to interpret our results. In order to present the result, we uploaded a new Figure as (shown as Additional files 3 and 4) which combined the Group magnesium in the study and the Group single incision from the model experiment in our previous study [9]. Both of the two study were performed by doc Sun at the same condition. Maybe we have made over-interpretation of the data. It was afraid that such differences did not have clinically meaningful. In conclusion, the increased expression of NR_2_B phosphorylation in the spinal dorsal horn played an important role in the progress of RIH. Intrathecal administration of MgSO_4_ could dose-dependently diminish RIH via decreasing the amount of pNR_2_B in the spinal dorsal horn. This study offered the potential therapeutic opportunities of MgSO_4_ for the management of opioid induced pain.

## Additional files

Additional file 1: Data of PWTL and PWMT values in the study. (XLS 19 kb)
Additional file 2: Data of ratio of grey values by β-actin in the western blot. (XLS 19 kb)
Additional file 3: The PWTL and PWMT tests in the previous model tests from the referred literature (A and B) and the ones after the NMDA antagonists therapy in this study (C and D). (XLS 18 kb)
Additional file 4: Data of PWTL and PWMT values in the previous model tests from the referred literature. (TIF 812 kb)

## Abbreviations

NMDA: N-methyl-D-aspartate
PWMT: Paw withdrawal mechanical thresholds
PWTL: Paw withdrawal thermal latency
RIH: Remifentanil-induced hyperalgesia
