# Supplementary figures and images for: Magnesium sulphate attenuate remifentanil-induced postoperative hyperalgesia via regulating tyrosine phosphorylation of the NR2B subunit of the NMDA receptor in the spinal cord
Source: BMC Anesthesiol. 2017 Feb 21;17:30. doi: 10.1186/s12871-017-0325-3 (PMC5320626; doi:10.1186/s12871-017-0325-3)

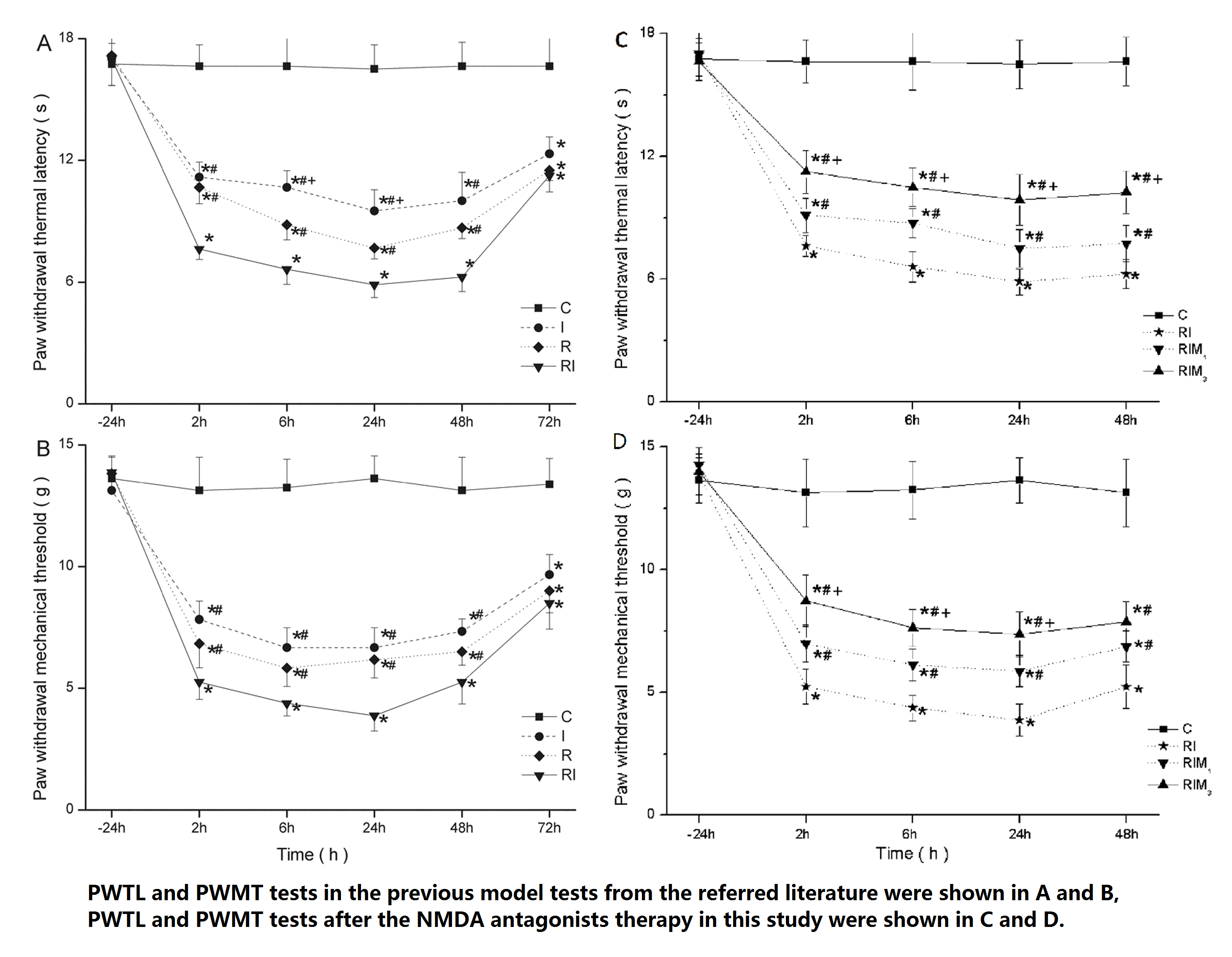

Supplement: Additional file 4: — Data of PWTL and PWMT values in the previous model tests from the referred literature. (TIF 812 kb) [file 12871_2017_325_MOESM4_ESM.tif]
